# Gender role attitudes and work–family conflict: A multiple mediating model including moderated mediation analysis

**DOI:** 10.3389/fpsyg.2022.1032154

**Published:** 2022-12-22

**Authors:** Gongxing Chen, Jiamiao Zhang, Yingying Hu, Yuan Gao

**Affiliations:** ^1^College of Education for the future, Beijing Normal University at Zhuhai, Zhuhai, China; ^2^Center for Mental Health, Guangxi Vocational College of Water Resources and Electric Power, Nanning, China; ^3^School of Psychology, Central China Normal University, Wuhan, China; ^4^International College, Beijing University of Agriculture, Beijing, China

**Keywords:** gender role attitudes, parental sacrifice, subjective well-being, work–family conflict, subjective socioeconomic status

## Abstract

With the fierce labor market competition, the family population’s size continues to expand, and the conflict between work and family requirements for individual roles becomes increasingly intense. Most studies focus on work–family conflict as an antecedent variable, and few studies use work–family conflict as an outcome variable. This study aimed to explore the underlying mechanism of the relationship between gender role attitudes and work–family conflict. Two models were tested using conditional process analysis for testing direct and indirect effects on a sample of 324 employees: A serial multiple mediation model, and the multiple mediation model including the moderating role of education level and subjective socioeconomic status. The results suggested that (1) gender role attitudes significantly and positively predicted work–family conflict. (2) Parental sacrifice and subjective well-being played multiple mediating roles between gender role attitudes and work–family conflict. (3) Education level moderated the relationship between gender role attitudes and parental sacrifice, as evidenced by the fact that low education level amplified the positive predictive effect of gender role attitudes on parental sacrifice. (4) Subjective socioeconomic status moderated the relationship between gender role attitudes and subjective well-being, suggesting that high subjective socioeconomic status amplified the negative predictive effect of gender role attitudes on subjective well-being. This work contributes to the understanding of the process underlying the relationship between gender role attitudes and work–family conflict, and to the literature reporting the possible moderated role of education level and subjective socioeconomic status on the influence outcomes of gender role attitudes. Theoretical and practical implications are also discussed.

## Introduction

Work and family are two major themes in people’s life. With the fierce competition in the labor market and the increase in family size, the conflict between work and family requirements for individual roles becomes increasingly intense. In recent years, there was increasing attention toward the phenomenon of work–family conflict, a form of inter-role conflict caused by incompatible work and family demands in some respect (Zhou et al., 2020; Yang et al., 2021; Miller et al., 2022). A study has found an unexpected positive effect of a higher amount of work–family conflict on the intention to have a second child because the mother of one child was still engaged in paid work and attaches great importance to family life and the birth of another child, which easily leads to conflicts between paid work and family responsibilities (Begall and Mills, 2011). It had also been found that work–family conflict was negatively related to satisfaction with life, and acted as a mechanism that helped to explain the relationship between autonomous motivation and satisfaction with life (Dominguez et al., 2021). At present, most studies focus on work–family conflict as an antecedent variable (Shreffler et al., 2010; Zhou et al., 2020; Yang et al., 2021), and few studies use work–family conflict as an outcome variable (Miller et al., 2022). A study examined the relationship between couples’ work–family arrangement and individuals’ perceived work–family conflict (WFC), considering individuals’ attitudes towards gender roles and national gender culture in 37 countries, and showed that individuals with egalitarian gender attitudes and an egalitarian work–family arrangement experience less WFC (Bornatici and Heers, 2020). In addition, the discordance between husband and wife in gender role attitudes would increase the role burden of the wife and further lead to family–work conflict (Hu et al., 2021). Research on the direct relationship between gender role attitudes and work–family conflict receives consistent attention (Hatchman, 2010; Yucel and Chung, 2021), whereas studies on the mechanism underlying this relationship are very scant. As a country with thousands of years of farming civilization and Confucian cultural tradition, China’s traditional gender role attitudes are engraved with the strong role concept that men farm and women weave, men work outside and women work inside (Liu et al., 2021). Therefore, the mechanisms underlying gender role attitudes to work–family conflict in this sociocultural context are worth exploring in depth. It may help guide scholars who are developing interventions to reduce work–family conflict and its interference.

## Theoretical framework

### Gender role attitudes and work–family conflict

According to the theory of conservation of resources, since the resources of individuals are limited, the resources available for another role will be reduced when spent on one role (Scafuri Kovalchuk et al., 2019). Therefore, when faced with the coexistence of two or more roles, it will be difficult for individuals to obey one role, which may lead to role conflict (Luhaorg and Zivian, 1995). Work–family conflict is a role conflict that arises from the incompatibility between the role pressures in the work domain and the family domain (Yucel and Chung, 2021). This study will discuss work–family conflict based on the theory of the conservation of resources. Several studies have shown that the impact of daily work stressors may hurt family dynamics (Taris et al., 2005; Clark et al., 2016). Another study has supported the relationship between work–family conflict and burnout, that is, employees who have experienced work–family conflict will exhaust energy and increase the risk of job burnout (Lizano and Mor Barak, 2012). Gender role attitudes refer to an individual s’ perceptions of gender social role norms, behavioral patterns, and the attitudinal tendencies held by them (Hatchman, 2010), and thus different perceptions and attitudes will be generated for different social role divisions. China’s traditional concepts of gender roles can imprison women in families, who are responsible for housework and child-rearing, while men work outside to support the family (Liu et al., 2021). However, in the real world, men and women need to share the responsibility of “providing for the family” and “taking care of the family,” and men with traditional gender role attitudes will feel the intrusion of the family on work, while women will feel the intrusion of work on the family more. Accordingly, we put forward our first hypothesis.

*Hypothesis H1*: A direct and positive relationship between gender role attitudes and work-family conflict may exist: Traditional gender role attitudes predict higher work-family conflict.

### Gender role attitudes, parental sacrifice, and work–family conflict

Parental sacrifice refers to the process of parents giving up their personal needs for the sake of their children’s educational needs and reflects the contribution of parents to their children’s education (Leung and Shek, 2011).

The sex-role theory (Bem, 1974) holds that femininity is related to expressiveness, while masculinity is related to instrumentality (Spence, 1993). Mothers may adopt a more emotional parenting style, while fathers may adopt a more goal-oriented approach (Russell et al., 1998). The role theory of cultural perspectives suggests that the role and practice of parents are determined by culture, and culture is historical development and traditional acceptance (Shek, 2002). For example, in the Chinese Confucian philosophy, fathers should assume the responsibility of training and supervising children’s behavior, while mothers, as caregivers, are responsible for maintaining child care and family management. Based on the cultural perspective of role theory, individuals with traditional gender role attitudes are more inclined to have more parental sacrifices in educating their children (Leung and Shek, 2012). In Chinese culture, parenthood connotes “taking responsibility” for one’s children and “making sacrifices for the benefit of the children” (Lam, 2005). Cultural meanings embedded in a particular culture shape parents’ belief systems and parental goals, which in turn instill and guide parental behavior (Padmawidjaja and Chao, 2010). A study found that the sacrifice made in one life field to deal with the affairs in another field will aggravate the conflict between work and family, and reduce the sense of happiness and satisfaction (Houlfort et al., 2022). Stimulus Organism Response (SOR) refers to a series of psychological and physiological reactions produced by human organisms under the action of internal and external stimuli (Mehrabian and Russell, 1974; Li et al., 2021a). As the internal psychological stimulus of individuals, gender role attitude may be used to cause work–family conflict through the level of parental sacrifice of the individual, that is, how to allocate limited resources (including funds, time, and energy) to the needs of children’s education and personal development. Thus, we hypothesize the mediating role of parental sacrifice in the relationship between gender role attitudes and work–family conflict.

*Hypothesis H2*: Parental sacrifice may mediate the relationship between gender role attitudes and work-family conflict.

### Gender role attitudes, subjective well-being, and work–family conflict

Subjective well-being is a psychological state, which is an individual’s satisfaction with his psychological state and the overall assessment of the positive and negative emotions he has experienced (Li et al., 2021b). Subjective well-being also reflects their social functioning and adaptation status (Zhang et al., 2022). While a study tended to examine subjective well-being as an outcome variable, with the development of positive psychology, researchers have found that subjective well-being can also serve a potential functional role for success in various areas of life (Xiao and Huang, 2022). Subjective well-being would be regarded as a dynamic energy system of individuals in the face of pressure or possible adversity, and it can form a good psychological environment for individuals to realize the hostile world (Shmotkin, 2005). As research has found, subjective well-being hurts adolescents’ Internet addiction behavior. When the happiness of individuals decreases, they will experience strong negative emotions, and their negative emotions can positively predict their smartphone addiction behavior (Li et al., 2021b). Research showed that individuals with traditional gender role attitudes are more likely to have greater psychological distress and cause work–family conflict (Hajar et al., 2010; Bornatici and Heers, 2020; Hu et al., 2021). Therefore, individuals would help themselves cope with life stress, such as role conflict, by improving subjective well-being, thus reducing work–family conflict. Accordingly, we hypothesize the mediating role of subjective well-being in the relationship between gender role attitudes and work–family conflict.

*Hypothesis H3*: Subjective well-being may mediate the relationship between gender role attitudes and work-family conflict.

### Gender role attitudes, parental sacrifice, subjective well-being, and work–family conflict

Although A study has found that prosocial behavior can increase personal and relationship well-being, sacrifice as a particularly costly prosocial behavior, will hurt the happiness of givers (Righetti et al., 2021). Some studies have considered that four different aspects of sacrifice (i.e., willingness to sacrifice, behavioral sacrifice, satisfaction with sacrifice, and costs of sacrifice) have different associations with well-being (Righetti et al., 2020). Specifically, an individual’s willingness to sacrifice was positively associated with their own personal and with their partner’s relationship well-being. However, behavioral sacrifice was negatively associated with own personal well-being. Satisfaction with sacrifice was positively associated with individual and partner well-being. Costs of sacrifice were negatively related to one’s personal and the partner’s relationship well-being.

According to the conservation of resources theory, resources will be lost. When an individual’s resources are limited, if resources are spent on one role, the resources available for another role will be reduced (Scafuri Kovalchuk et al., 2019). Individuals with traditional gender role attitudes are more inclined to have more parental sacrifices in educating their children (Leung and Shek, 2012). In that case, the individual’s parental sacrifice will reduce the subjective well-being, and further aggravate the work–family conflict. According to this literature, we hypothesized a mediation effect of parental sacrifice and subjective well-being in the relationship between gender role attitudes and work–family conflict.

*Hypothesis H4*: Parental sacrifice and subjective well-being would mediate the relationship between gender role attitudes and work-family conflict.

### Gender role attitudes, education level, and parental sacrifice

From the perspective of socialization, individuals who have received higher education will be exposed to more free ideas, raising their expectations of gender equality, enabling them to question traditional values, and often take a positive attitude towards their rights and interests (Cheng and Hsu, 2020), especially for women. Women with higher education will envision less housework and more equal family gender relations (Fetterolf and Eagly, 2011). Compared with women with lower education, women with higher education are more likely to expect a more equal division of labor in the family, even if their income levels are similar (Cunningham et al., 2005). In the case of deep-rooted traditional family norms and slow social adaptation to the improvement of the equal status of men and women, individuals with high education are more likely to struggle or even resist, and thus show a lower level of parental sacrifice. In addition, according to the family investment model, individuals with high education levels are more capable of investing high-quality resources in their children, and they may achieve the goal of educating their children without sacrificing themselves (Conger and Donnellan, 2007). Therefore, in the present study, we hypothesize a moderated effect of education level on the relationship between gender role attitudes and parental sacrifice. More particularly, we envisage that the higher the education level, the weaker the relationship between their gender role attitudes and parental sacrifice.

*Hypothesis H5*: Education Level would negatively moderate the relationship between gender role attitudes and parental sacrifice: The gender role attitudes and parental sacrifice relationship are weaker in high education level relationships than in low education level relationships.

### Gender role attitudes, subjective socioeconomic status, and subjective well-being

Subjective socioeconomic status (SSES) is an individual’s self-perception and evaluation of his or her economic position in a social group (Wang et al., 2022). It was found that subjective socioeconomic status can positively predict subjective well-being (Yang and Pang, 2018). A study on the elderly has found that subjective socioeconomic status was a robust positive predictor of subjective well-being (George, 2010). The psychological orientation account suggests Individuals of different subjective socioeconomic statuses will exhibit different psychological behavior patterns (Reissman and Kohn, 1970). Individuals with higher subjective socioeconomic status are more optimistic about their resources and opportunities, more likely to develop self-centered habits and expectations of control and ignore the situational constraints on their choices and behaviors. Individuals with lower subjective socioeconomic status perceive their limited resources and opportunities, which limits their choices and behaviors (Karney, 2021). Therefore, under different subjective socioeconomic statuses, there are differences in the influence process of individual gender role attitudes on subjective well-being. Individuals with high subjective socioeconomic status are more likely to amplify their feelings because they have stronger self-centered habits and control expectations. In the present study, we hypothesize the moderated effect of subjective socioeconomic status in the relationship between gender role attitudes and subjective well-being. Specifically, we envisage the higher their subjective socioeconomic status, the stronger the relationship between their gender role attitudes and subjective well-being.

*Hypothesis H6*: Subjective socioeconomic status would positively moderate the relationship between gender role attitudes and subjective well-being: The gender role attitudes and subjective well-being relationship is stronger in high subjective socioeconomic status relationships than in low subjective socioeconomic status relationships.

**Figure 1:**
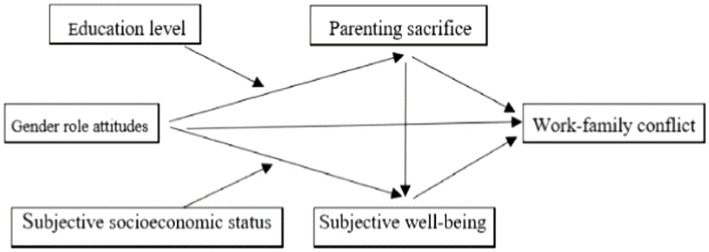
The relationship between gender role attitudes, parental sacrifice, subjective well-being, and work–family conflict.

Based on the above arguments, the theoretical model is proposed (Figure 1).

## Materials and methods

### Participants and procedures

This research was approved by the Research Ethics Committee of Guangxi Vocational College of Water Resources and Electric Power. Four researchers trained professionally recruited survey participants through snowball sampling using acquaintance social networks. Our research team developed an online questionnaire on the WJX of a professional survey company in China.^1^ The researchers distributed the online questionnaire to the participants through the WeChat group, and the participants took about 20 min to complete the questionnaire. Our participants are social workers from high, middle, and low economic development areas in China, distributed in Guangdong, Jiangxi, and Guangxi provinces. Considering the research theme of work–family conflict, women are more likely to be recruited from the perspective of gender distribution.

Before collecting data, we provided participants with written informed consent, including a brief description of the research project, and told participants that any information they provided would be confidential and that their names would not be recorded. The study was conducted by the Declaration of Helsinki, and the participants were voluntary and rewarded with personal feedback (test scores and interpretation of results). Personal feedback can encourage them to answer the questionnaire seriously and faithfully. In addition, participants’ answers will only be used for research purposes, and they can stop participating at any time with impunity.

The research design is a cross-sectional design. A total of 324 questionnaires were distributed and returned; 323 (99.69%) participants provided valid responses. One participant responded consistently to all items and was considered invalid data. The sample consisted of 225 women (69.66%) and 98 men (30.34%). Their age ranged from 19 to 52 years (M = 35.35; SD = 4.93)0.13 participants (4.02%) with an education level of high school or below, 24 participants (7.43%) with junior college, 119 participants (36.84%) with a bachelor’s degree, 135 participants (41.80%) with a master’s degree and 32 participants (9.91%) with doctor’s degree. The subjective socioeconomic status of 11 participants (3.41%) was far lower than the average level, 38 participants (11.76%) were lower than the average level, 169 participants (52.32%) were higher than the average level, 100 participants (30.96%) were higher than the average level, and 5 participants (1.55%) were far higher than the average level.

### Measures

#### Gender role attitudes

The gender role attitudes scale developed by the Renmin University of China in CGSS 2015 (China General Social Survey) was used to define gender role attitudes according to two dimensions, modern and traditional, focusing on the sense of equalization of gender role attitudes, including five dimensions of family and career, competence traits, self-worth, labor and employment, and family division of labor. Using the 7-point Likert scale, the higher the score, the more traditional the gender role attitudes. In this study, Cronbach’s alpha coefficient for the scale was 0.74. The results of confirmatory factor analysis are as follows: χ^2^/df = 1.73, RMSEA = 0.05, SRMR = 0.03, CFI = 0.99, and TLI = 0.98, indicating that the model fitted well.

#### Parental sacrifice

The parental sacrifice scale developed by Leung and Shek, (2011) was used, which is appropriate for China, and the scale has 23 items in 5 dimensions, and the 5 dimensions strive for financial resources, time spent on the educational needs of children, restructuring of daily routine, the sacrifice of lifestyle and aspiration, shielding of worries. a 5-point Likert scale from 1 (strongly disagree) to 5 (strongly agree) is used, and the higher the score, the greater the parental sacrifice (Leung and Shek, 2011). In this study, Cronbach’s alpha coefficient for the scale was 0.93. The results of confirmatory factor analysis are as follows: χ^2^/df = 4.09, RMSEA = 0.09, SRMR = 0.07, CFI = 0.86, and TLI = 0.85, indicating that the model fitted well.

#### Subjective well-being

The Index of Well-Being scale developed by Campbell (1976) was used. The scale consists of two components: the overall affective index and life satisfaction. The former consists of eight items with a weight of 1; the latter has only one item with a weight of 1.1. A 7-point Likert scale is used, with higher scores indicating higher subjective well-being (Campbell, 1976). In this study, Cronbach’s alpha coefficient for the scale was 0.93. The results of confirmatory factor analysis are as follows: χ^2^/df = 4.80, RMSEA = 0.08, SRMR = 0.05, CFI = 0.92, and TLI = 0.85, indicating that the model fitted well.

#### Work–family conflict

The Work–Family Conflict Scale was used to measure the level of work–family conflict. This scale was developed by Carlson et al. (2000). The scale has 18 items, and contains two dimensions: work interference with family and family interference with work. A 5-point Likert scale is used, with higher scores indicating higher work–family conflict (Carlson et al., 2000). In this study, Cronbach’s alpha coefficient for the scale was 0.92. The results of confirmatory factor analysis are as follows: χ^2^/df = 4.69, RMSEA = 0.09, SRMR = 0.06, CFI = 0.85, and TLI = 0.81, indicating that the model fitted well.

#### Education level

Based on the current situation of China’s national education and the classification of Chinese academic qualifications and degrees, the education levels used in our study include five categories: below high school, junior college, undergraduate, master, and doctorate. Score 1–5. The higher the score, the higher the education level.

#### Subjective socioeconomic status

Using the MacArthur Scale of subjective socioeconomic status, Participants were given an image of a ladder with 5 rungs and were asked to place themselves on a rung of the ladder that best represented their position in society. A higher rung indicated that the individual had a higher level of subjective socioeconomic status (Wang et al., 2022).

#### Physical health status

The physical health status variable was used to describe the participants’ physical health. It is divided into 5 grades, ranging from 1(Strongly unhealthy) to 5(Strongly healthy). The higher the score, the healthier the participants are.

### Data analysis

SPSS22.0 and Mplus8.3 were used to manage and analyze the data. SPSS22.0 was used mainly for preliminary data processing, descriptive statistics, reliability test, and correlation analysis. Whereas Mplus8.3 was used for moderated mediation model analysis through conditional process analysis based on ordinary least squares (OLS) regression using a bootstrapping technique (Preacher and Hayes, 2004). Specifically, physical health status was inserted in the model as a control variable.

## Results

### Common method variance tests

The data in this study were collected through self-report, which may have common method variance(CMV). Some methods had been taken to reduce this problem, such as reverse coding of items, anonymous answers, and balancing the order of items. Meanwhile, Harman’s single-factor test was performed in SPSS to determine the influence of CMV on the study results. Moreover, we conducted a test on the analysis result of the non-rotating factor by including all the study’s variables. The results showed that a total of 11 factors exhibited characteristic roots greater than 1, with the variance interpretation rate of the first factor being 20.83% (less than 50%; Harris et al., 2009), which indicated that the CMV has a slight impact on the present study results.

### Descriptive statistics and the correlation among the studied variables

The results of the descriptive statistics and correlation analysis of each variable are shown in Table 1, and physical health status was used as a control variable during the subsequent statistical analysis because it was associated with work–family conflict. The reliability coefficients expressed by Cronbach α ranged from 0.74 (gender role attitudes) to 0.93 (physical health status), indicating satisfactory internal reliability for all variables.

**Table 1:** Descriptions, inter-correlations, and reliabilities of the study variables.

Variables	Mean	SD	1	2	3	4	5	6
1. gender role attitudes	2.000	0.780	**(0.74)**					
2. work–family conflict	2.776	0.669	0.208^**^	**(0.92)**				
3. parental sacrifice	4.210	0.849	0.171^**^	0.265^**^	**(0.93)**			
4. subjective well-being	1.161	0.224	−0.116^*^	−0.319^**^	−0.004	**(0.93)**		
5. Education Level	3.460	0.916	−0.102	−0.022	−0.155^**^	0.065		
6. subjective socioeconomic status	3.150	0.777	0.045	−0.095	−0.033	0.145^**^	0.196^**^	
7. physical health status	3.410	0.826	0.067	−0.156^**^	−0.013	0.130^**^	0.039	0.129^*^

*n* = 323; Gender was coded as 1 = men and 2 = women; ** *p* < 0.01; * *p* < 0.05; Cronbach’s alphas are in the diagonal in bold.

### Serial multiple mediation model

The serial Multiple mediating effects of parental sacrifice, subjective well-being between gender role attitudes, and work–family conflict were tested using Mplus8.3 with physical health status as a control variable (model 1), and the fitted indicators were χ^2^/df = 3.30, RMSEA = 0.08, CFI = 0.95, TLI = 0.76, SRMR = 0.04. The bias-corrected percentile Bootstrap method test was further applied, with 1,500 replicate samples and 95% confidence intervals calculated. Gender role attitudes significantly and positively predicted work–family conflict (β = 0.143, 95% Bootstrap CI [0.033, 0.052]), and hypothesis H1 was supported. The mediating effect of parental sacrifice was significant (95% Bootstrap CI [0.013, 0.080]), with a mediating effect value of 0.041, accounting for 18.89% of the total effect of each variable, and hypothesis H2 was supported. The mediating effect of subjective well-being was significant (95% Bootstrap CI [0.004, 0.073]), with a mediating effect value of 0.034, accounting for 15.67% of the total effect of each variable, hypothesis H3 was supported. The serial mediating effect of parental sacrifice and subjective well-being was not significant, and H4 was not supported by the evidence (95% Bootstrap CI [−0.012, 0.005]). Thus, parental sacrifice and subjective well-being were modeled as multiple mediating models between gender role attitudes and work–family conflict (model 2). Model 2 was tested and found that the fitting degree (χ^2^/df = 2.23, RMSEA = 0.06, CFI = 0.96, TLI = 0.87, SRMR = 0.04) was better than model 1, which further verified the multiple mediating models between gender role attitudes and work–family conflict.

### Moderated mediation model

**Figure 2:**
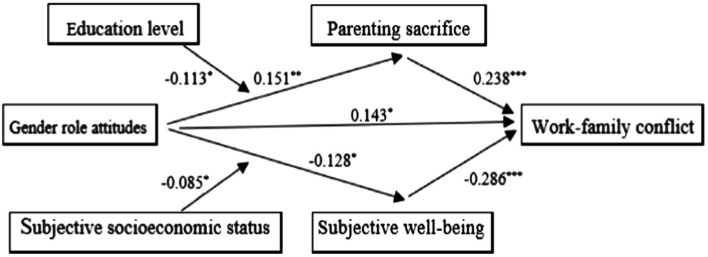
A multiple mediating model including moderated mediation analysis.

The data were first standardized to test the moderating role of education level in the process of gender role attitudes influencing work–family conflict through parental sacrifice and the moderating role of subjective socioeconomic status in the process of gender role attitudes influencing work–family conflict through subjective well-being, by Mplus8.3, controlling for physical health status. The results of the test are shown in Table 2. The result shows that the model is a fit. The interaction between gender role attitudes and education level was significant (β = −0.113, *t* = −2.176, *p* < 0.05), indicating that education level plays a moderating role in the relationship between gender role attitudes and parental sacrifice. The interaction between gender role attitudes and subjective socioeconomic status was significant (β = −0.085, *t* = 2.026, *p* < 0.05), indicating that subjective socioeconomic status plays a moderating role in the relationship between gender role attitudes and subjective well-being. Therefore, this study validated multiple mediating models including moderated mediation analysis, as shown in Figure 2. The values of the mediating effect of parental sacrifice and subjective well-being between gender role attitudes and work–family conflict and their 95% bootstrap confidence intervals, based on the mean and mean ± 1 standard deviation of education levels and subjective socioeconomic status, were divided into three levels as shown in Tables 3, 4.

**Table 2:** Results of conditional process analysis.

	PASA			SWB			WFC		
	β	SE	t	β	SE	t	β	SE	t
GRA	0.151	0.056	2.683^**^	−0.128	0.055	−2.318^*^	0.143	0.057	2.503^***^
EL	−0.132	0.050	−2.659^**^						
GRA × EL	−0.113	0.052	−2.176^*^						
PASA							0.238	0.058	4.082^***^
SSES				0.154	0.051	2.984^**^			
GRA × SSES				−0.085	0.042	−2.026^*^			
SWB							−0.286	0.054	−5.249^***^
PHS							−0.152	0.059	−2.575^*^
R^2^		0.068			0.049			0.204	
F		10.945^***^			13.223^***^			12.845^***^	
**χ** ^**2**^					18.85				
df					11				
p					0.064				

GRA = gender role attitudes; WFC = work–family conflict; PASA = parental sacrifice; SWB = subjective well-being; EL = Education Level; SSES = subjective socioeconomic status; PHS = physical health status; df = degrees of freedom.

**Table 3:** The mediating effect of parental sacrifice between gender role attitudes and work–family conflict at different education levels.

Education levels	Effect	Boot SE	Boot LLCI	Boot ULCI
Mean-1SD	0.063	0.022	0.026	0.116
Mean	0.036	0.015	0.010	0.072
Mean + 1SD	0.009	0.020	−0.029	0.048

**Table 4:** The mediating effects of subjective well-being between gender role attitudes and work–family conflict at different subjective socioeconomic status.

Subjective socioeconomic status	Effect	Boot SE	Boot LLCI	Boot ULCI
Mean-1SD	0.013	0.019	−0.022	0.055
Mean	0.038	0.018	0.007	0.079
Mean + 1SD	0.062	0.025	0.021	0.124

**Figure 3:**
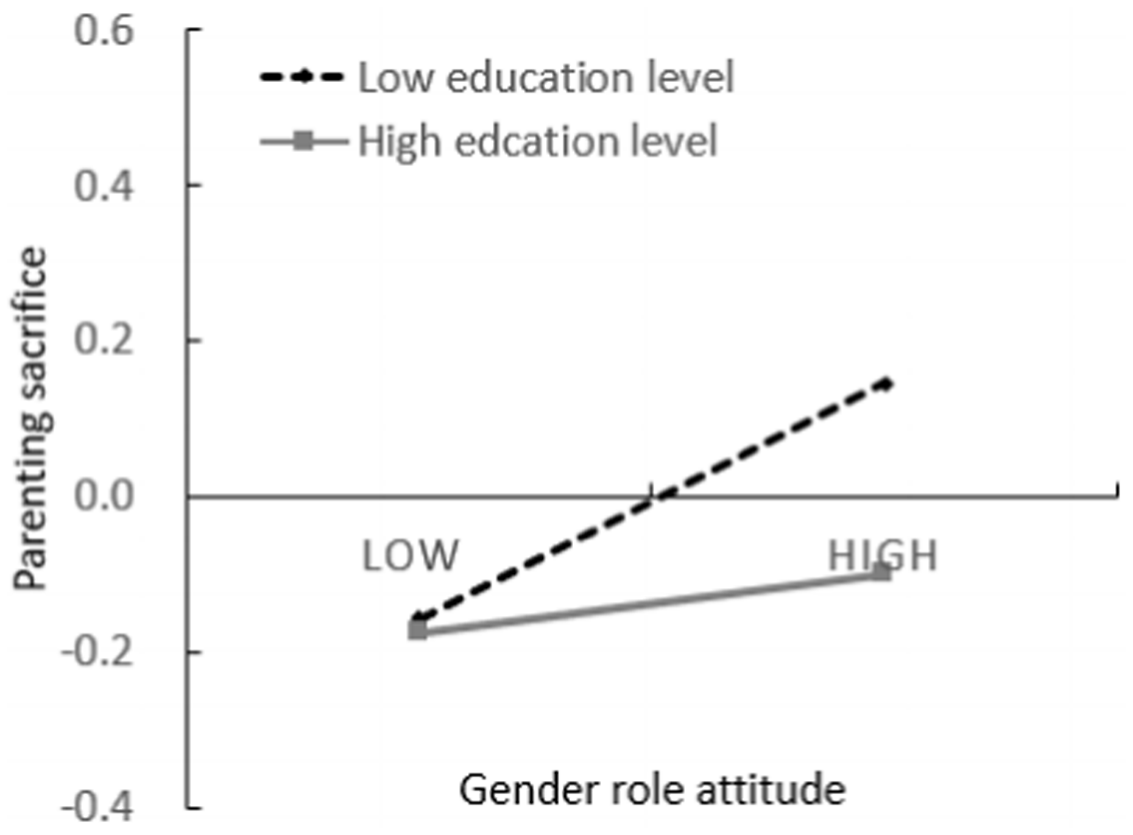
Interactive effects of gender role attitude and education levels on parental sacrifice.

**Figure 4:**
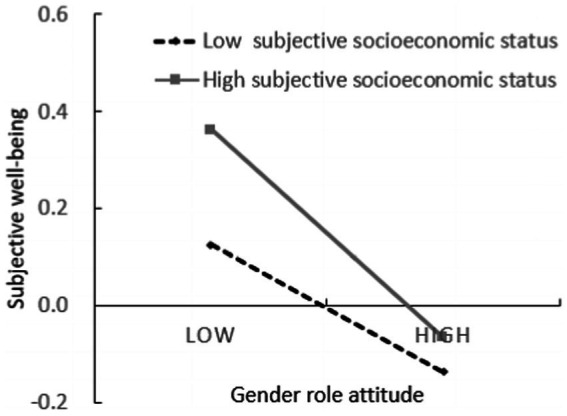
Interactive effects of gender role attitude and subjective economic status on subjective well-being.

To further analyze the essence of the moderating effect, the z scores of education level and subjective economic status were divided into groups with 1 (high education level group or high subjective economic status group) and - 1 (low education level group or low subjective economic status group), and simple slope test was carried out. As shown in Figures 3, 4. The results showed that when the education level was low, the gender role attitudes had a significant positive predictive effect on parental sacrifice (simple slope = 0.265, *t* = 3.728, *p* < 0.001); When the education level is high, the gender role attitude has no significant predictive effect on the parental sacrifice (simple slope = 0.038, *t* = 0.459, *p* > 0.05). Thus, hypothesis H6 was supported. When the level of subjective socio-economic status is low, gender role attitude has no significant predictive effect on subjective well-being (simple slope = −0.047, *t* = −0.724, *p* > 0.05); When the level of subjective socio-economic status is high, gender role attitude has a significant negative predictive effect on subjective well-being (simple slope = −0.216, *t* = −2.882, *p* < 0.01). Thus, hypothesis H6 was supported.

## Discussion

The aim of the current study was twofold: (1) examining the mediating role played by both parental sacrifice and subjective well-being in the relationship between gender role attitudes and work–family conflict; (2) investigating the moderating role of education level and subjective socioeconomic status in the relationship between gender role attitudes and work–family conflict. Regarding the first aim, the results supported the multiple mediation model. This contributes to the literature on the relationship between gender role attitudes and work–family conflict by highlighting the possible process of work–family conflict caused by different gender role attitudes. This study suggested that traditional gender role attitudes positively predict work–family conflict, which is consistent with the findings of a study (Bornatici and Heers, 2020), and supports role conflict theory (Luhaorg and Zivian, 1995; Yucel and Chung, 2021). Gender role attitudes can indirectly predict work–family conflict through parental sacrifice, and the more traditional the gender role attitudes, the higher the parental sacrifice, and thus the higher the work–family conflict, in line with another study (Leung and Shek, 2011). In Chinese traditional culture, parents with traditional gender role attitudes often think that the happiness of their children is the greatest value of their lives, and education is an important way to make their children happy in life. They are more willing to make sacrifices for their children’s education. The process of upbringing sacrifice is a process in which individuals play the role of “parents,” which requires time and energy. According to the theory of role conflict, it is difficult for an individual to assume another role when he plays one role (Luhaorg and Zivian, 1995). Therefore, when individuals allocate their limited time and energy in terms of parental sacrifice, it may predict higher work–family conflict. This study suggested that gender role attitudes indirectly predict work–family conflict through subjective well-being, and the more traditional the gender role attitude is, the lower subjective well-being is, which predicts the higher work–family conflict. A study also found that individuals with more traditional attitudes (traditionalism and paid employment) towards gender roles are more likely to show low-level subjective well-being (Sweeting et al., 2014). The results support that subjective well-being will be regarded as a dynamic energy system when individuals face pressure or possible adversity, which can form a good psychological environment for individuals to cope with the world (Shmotkin, 2005).

It is worth mentioning that parental sacrifice has no significant predictive effect on subjective well-being, so H4 was not supported by the evidence. This study did not distinguish the individual’s parental sacrifice from the type, which may be the reason why parental sacrifice cannot significantly predict subjective well-being, and this is a direction that can be further studied in the future. Through a meta-analysis of sacrifice and personal well-being, researchers found that sacrifice includes four different aspects: sacrifice willingness, behavior sacrifice, sacrifice satisfaction, and sacrifice cost, which have different associations with well-being (Righetti et al., 2020). Sacrifice willingness refers to the willingness or motivation to give up personal interests for the sake of a partner or relationship. Behavior sacrifice refers to whether the sacrifice occurs. Sacrifice satisfaction refers to the degree to which people are satisfied with the sacrifice, and sacrifice cost refers to the degree to which people realize that their sacrifice has brought costs. Specifically, individuals’ willingness to sacrifice is positively correlated with their own personal and their partner’s relationship well-being; Behavior sacrifice is negatively correlated with personal well-being; The satisfaction of sacrifice is positively correlated with the happiness of individual and partner; The cost of sacrifice is negatively related to a person’s personal and a partner’s relationship well-being.

As for the second purpose of this study, the hypotheses were also supported by the results. The low education level magnifies the role of gender role attitude in promoting parental sacrifice, that is, for individuals with a low education level, the more traditional the gender role attitude is, the higher the parental sacrifice is. For individuals with high education levels, gender role attitude has no significant predictive effect on parental sacrifice. According to the family investment model, individuals with high education levels are more capable of investing high-quality resources in their children, and they may achieve the goal of educating their children without sacrificing themselves (Conger and Donnellan, 2007); In addition, individuals with high education level have a higher level of knowledge, culture and legal awareness, are more inclined to freedom and independence and are more likely to expect their children to be more independent in the process of raising their children (Fetterolf and Eagly, 2011), so they show lower sacrifice in raising their children. The moderating effect of subjective socioeconomic status is that high subjective socioeconomic status amplifies the negative predictive effect of gender role attitude on subjective well-being, and this negative predictive effect is only reflected in individuals with high subjective socioeconomic status. For individuals with low subjective socioeconomic status, gender role attitude has no significant predictive effect on subjective well-being. It supports the psychological orientation account theory that individuals with different subjective socioeconomic statuses will show different psychological behavior patterns (Reissman and Kohn, 1970; Karney, 2021). Individuals with a higher level of subjective socioeconomic status take for granted the resources and opportunities they have, tend to have a self-focus, and expect to maintain control, neglecting the role of contextual constraints on their intention and behavior. In contrast, the life outcomes of individuals with lower subjective socioeconomic statuses are often controlled by external forces, and they are more sensitive to the social environment and the interdependence of individuals in the environment (Manstead, 2018). Thus, the subjective well-being of individuals with low subjective socioeconomic status will be more influenced by social environment than by individual gender role attitudes. On the contrary, individuals with high subjective socioeconomic status, are more concerned about their inner state, so their subjective well-being is more influenced by their attitudes toward gender roles.

### Implications

Despite a large number of studies examining the roles of gender role attitudes, parental sacrifice, subjective well-being, education level, and subjective socioeconomic status in work–family conflict, how these factors interplay in work–family conflict is rarely studied. Our study contributes to the literature by integrating these factors in a single model and providing a better understanding of the mechanisms of how these factors operate together in affecting work–family conflict. The study found that the multiple mediating roles of parental sacrifice and subjective well-being, as well as the moderating role of education level and subjective socioeconomic status, have important theoretical implications.

The present study provides ideas for the prevention of work–family conflict and has important practical implications. First, gender role attitudes can directly and significantly predict work–family conflict. Therefore, Under the trend of social development of gender equality, gender role attitudes should also keep pace with equality to reduce work–family conflict. Second, gender role attitudes can predict work–family conflict through mediation between parental sacrifice and subjective well-being, indicating that both these variables may be the key factors influencing work–family conflict. Second, gender role attitudes can predict work–family conflict through mediation between parental sacrifice and subjective well-being, indicating that both these variables may be the key factors influencing work–family conflict. In considering how to balance work-family relationships, the focus can be on how individuals can effectively allocate psychological resources and enhance subjective well-being. Third, education level and subjective socioeconomic status played a moderating role, expanding previous research. The results suggest that individuals with a low education level of traditional gender role attitudes should be given reasonable guidance on parental sacrifice, and individuals with high subjective socioeconomic status can be guided by actively inducing an equal gender role attitude change to enhance subjective well-being and ultimately reduce work–family conflict.

### Limitations

We have to acknowledge some limitations in this study. First, although we theoretically gender role attitudes identified as the antecedent of work–family conflict, we cannot infer a causal relationship between gender role attitude and work–family conflict with this cross-sectional design, which can be tested in future longitudinal or experimental studies. Second, the variables in this study were assessed with self-reported data, but fortunately, we statistically tested the common method bias to be a slight impact. Future studies may consider multiple data sources. Third, this study was conducted by social workers in China. Whether the findings can be extended to other countries needs further examination. In addition, this study did not distinguish the individual’s parental sacrifice from the type, which may be the reason why parental sacrifice cannot significantly predict subjective well-being, and this is a direction that can be further studied in the future.

## Data availability statement

The original contributions presented in the study are included in the article/supplementary material, further inquiries can be directed to the corresponding author.

## Author contributions

GC and YG: conceptualization and project administration. JZ and YH: data collection. GC, YG, and JZ: formal analysis and writing of the original draft. GC, YH, and JZ: methodology. All authors contributed to the article and approved the submitted version.

## Funding

This work was supported by Guangxi University Young and Middle-aged Teachers’ Scientific Research Basic Ability Promotion Project (Grant no. 2022KY1083). Teaching Reform and Scientific Research Project of Guangxi Vocational College of Water Resources and Electric Power (Grant no. 2021yb34).

## Conflict of interest

The authors declare that the research was conducted in the absence of any commercial or financial relationships that could be construed as a potential conflict of interest.

## Publisher’s note

All claims expressed in this article are solely those of the authors and do not necessarily represent those of their affiliated organizations, or those of the publisher, the editors and the reviewers. Any product that may be evaluated in this article, or claim that may be made by its manufacturer, is not guaranteed or endorsed by the publisher.

## Footnotes

^1^ https://www.wjx.cn/
